# The Efficacy and Safety of Immune Checkpoint Inhibitors in Patients With Cancer and Preexisting Autoimmune Disease

**DOI:** 10.3389/fonc.2021.625872

**Published:** 2021-02-22

**Authors:** Hui Tang, Jianfeng Zhou, Chunmei Bai

**Affiliations:** Department of Medical Oncology, Peking Union Medical College Hospital, Chinese Academy of Medical Sciences and Peking Union Medical College, Beijing, China

**Keywords:** autoimmune disorder, solid tumors, immunotherapy, immune-related adverse events, PD-1, CTLA-4

## Abstract

Immune checkpoint inhibitor (ICI) is a revolutionary breakthrough in the field of cancer treatment. Because of dysregulated activation of the immune system, patients with autoimmune disease (AID) are usually excluded from ICI clinical trials. Due to a large number of cancer patients with preexisting AID, the safety and efficacy of ICIs in these patients deserve more attention. This review summarizes and analyzes the data regarding ICI therapy in cancer patients with preexisting AID from 17 published studies. Available data suggests that the efficacy of ICIs in AID patients is comparable to that in the general population, and the incidence of immune-related adverse events (irAEs) is higher but still manageable. It is recommended to administer ICIs with close monitoring of irAEs in patients with a possibly high benefit-risk ratio after a multidisciplinary discussion based on the patient’s AID category and severity, the patient’s tumor type and prognosis, alternative treatment options, and the patient’s intention. Besides, the prevention and management of irAEs in AID patients have been discussed.

## Introduction

The approved immune checkpoint inhibitors (ICIs) mainly involve several immune checkpoint–directed antibodies targeting cytotoxic T lymphocyte–associated antigen 4 (CTLA-4) and programmed cell death protein 1 (PD-1) or its ligand, programmed cell death ligand 1 (PD-L1). CTLA-4 inhibits an immune response in several ways, including hindering autoreactive T-cell activation at a proximal step in the immune response, typically in lymph nodes (1, 2). In contrast, the PD-1 pathway regulates T cells at a later stage of the immune response, typically in peripheral tissues (3). Differing from traditional chemotherapy and targeted therapy, ICI can break the state of immune tolerance in the tumor microenvironment (TME) and activate the body’s anti-tumor immunity. Clinical trials of ICI therapy are in full swing, showing remarkable efficacy as well as fewer and milder adverse events compared to chemotherapy, and the indications for ICIs continue to expand across malignancies (4).

Autoimmune diseases (AIDs) represent a family of at least 80 diseases that share a common pathogenesis: an improper activation of the immune system attacking the body’s own organs (5). By increasing the activity of the immune system, ICI may result in immune-related adverse events (irAEs). Although the precise underlying mechanism is unknown, irAEs show many common clinical manifestations and pathophysiology features similar to AID (6). The role of PD-1 and CTLA-4 in autoantigen tolerance has been widely recognized (7), so ICI may exacerbate the damage done by one’s own immune system to AID patients and bring forth autoimmune inflammatory manifestations similar to or unrelated to the baseline AID. AID patients were usually excluded from clinical trials of ICI therapy due to the potential for increased toxicity (8).

AIDs may cause chronic inflammation and increase the risk of carcinogenesis (9–12). Cancer can also increase the risk of AIDs (13). The incidence of AIDs in lung cancer patients was reported to be as high as 14% to 25% (8), and some cancers show clinical features similar to AIDs (9, 14). Due to a large number of cancer patients with preexisting AID, the safety and efficacy of ICIs in these patients deserve more attention. Furthermore, there is a tendency toward immune activation in AID patients, so some clinicians proposed that AID patients may be likely to benefit more from ICI (15). At present, there is no reported prospective randomized controlled study on this issue. Whether the efficacy and safety of ICIs in patients with cancer and preexisting AID differ from that in the general population is unclear.

## Relationship Between Immune Checkpoints and AID

CTLA-4 signaling has been shown to be involved in the pathogenesis of many AIDs including rheumatoid arthritis (RA), systemic lupus erythematosus (SLE), multiple sclerosis (MS), and type-1 diabetes (T1D) (16). Polymorphisms in CTLA-4 gene were also associated with disease susceptibility to many AIDs like SLE, RA, MS, T1D, and so on (1). Human patients with heterozygous loss of function mutations in CTLA-4 developed widespread autoimmunity including autoimmune hepatitis, T1D, and arthritis (17). CTLA-4 gene deletion could lead mice to develop lymphoproliferative disease and die by 3–4 weeks of age (18). By contrast, mice with ablation of CTLA-4 expression during adulthood developed severe, but not fatal, autoimmunity (19). Preclinical models showed CTLA-4 blockade worsened autoimmune thyroiditis, with a more aggressive mononuclear cells infiltration in thyroid (20).

PD-1 signaling were also involved in the pathogenesis of many AIDs like autoimmune hepatitis, inflammatory bowel disease (IBD), SLE, myocarditis, and RA (21). Polymorphisms in PDCD1 gene were associated with disease susceptibility to many AIDs including SLE, RA, Graves’ disease, and so on (1). The engagement of Tregs with autoreactive B cells *via* the PD-1/L1 inhibitory axis can trigger B cells apoptosis and inhibit the production of autoantibody (22). In patients with RA, lymphocytes infiltrating the synovium commonly express PD-1, the synovial lining cells express PD-L1, and the number of PD-1-positive lymphocytes was significantly larger in RA than in osteoarthritis (23). In addition, the PD-L1 expression on synovial lining cells was positively related to the number of infiltrating T cells and Krenn’s synovitis score (23), indicating an important role of PD-1 pathway in RA. Notably, in non-obese diabetic mice, both anti–CTLA-4 and anti–PD-1 treatment can prevent anergy induction in islet antigen–specific T cells, but only PD-1/L1 blockade can reverse experimentally induced anergy, indicating a unique function for PD-1 signaling in maintaining T cells anergy (24).

Furthermore, the administration of ICI may conflict with the management of AIDs. For example, abatacept is a fusion protein comprising the extracellular domain of CTLA-4, that competitively blocks the T cells CD28-CD80 pathway signaling and improves the prognosis of RA (25). Therefore, in contrast, the use of ipilimumab which blockades CTLA-4 signaling may conflict with the management of RA.

## Literature Experience With ICIs in AID Patients

To evaluate the efficacy and safety of ICIs in patients with cancer and preexisting AID, we summarized the retrospective studies published before October 2020 (**Table 1**). Inclusion criteria was articles available in full text, published in English, and reporting safety or efficacy data on patients with preexisting AID and cancer treated with ICI. Further, case reports and review articles were excluded. After screening, 17 published studies were included, from which the following data were extracted: author, publication year, sample size, characteristics of AID, cancer and ICI type; the number and proportion of AID flares, newly developed irAEs, treatment discontinuation and response; survival time. The irAEs reported in these studies can be divided into two categories. The first type is the flare of preexisting AIDs, and the second type is the newly developed irAEs that does not have a clear causal link with preexisting AIDs. We refer to the two types collectively as total irAEs (TirAEs). Most studies included patients regardless of the treatment line, so caution should be exercised when comparing studies’ efficacy data with previous clinical trials.

**Table 1:** Data summary of cancer patients with preexisting autoimmune disease treated with immune checkpoint inhibitors.

Cancer	Target	Study	Main AID	N	Efficacy			Safety			
					ORR	mPFS (months)	mOS (months)	TirAEs (grade ≥3)	Preexisting AID flare (grade ≥3)	Newly developed irAEs (grade ≥3)	Treatment discontinuation
Melanoma	CTLA-4	Kähler (26)	Thyroiditis (37%), Ps (17%), RA (15%)	41	12%	NA	NA	44% (NA)	29% (NA)	29% (NA)	17% due to AID flare
Melanoma	CTLA-4	Johnson (27)	RA (20%), IBD (20%), Ps (17%)	30	20%	3.0	12.5	50% (NA)	27% (NA)	33% (33%)	NA
Melanoma	CTLA-4	Lee (28)	RA (100%)	8	50%	NA	NA	100% (63%)	75% (25%)	50% (50%)	63% due to TirAEs
Melanoma	PD-1	Menzies (15)	RA (25%), Ps (12%), Colitis (10%)	52	33%	6.2	NR	NA	38% (6%)	29% (10%)	4% and 8% permanent discontinuation due to AID flare and newly developed irAEs, respectively
Melanoma	PD-1	Gutzmer (29)	Thyroiditis (26%), RA (21%), Ps (16%)	19	32%	NA	NA	58% (16%)	42% (11%)	16% (5%)	None
NSCLC	PD-1	Yoneshima (30)	ANA positivity (100%)	18	28%	2.9	11.6	33% (11%)	NA	NA	11% due to TirAEs
NSCLC	PD-1	Leonardi (31)	Ps (25%), RA (20%), Thyroiditis (16%)	56	22%	NA	NA	55% (NA)	23% (4%)	38% (11%)	0% and 14% permanent discontinuation due to AID flare and newly developed irAEs, respectively
Urological cancers	PD-1	Loriot (32)	Ps (43%), Thyroiditis (17%), RA (11%)	35	11%	8.2	4.4	46% (14%)	11% (6%)	NA	14% due to TirAEs
Urological cancers	PD-1 or CTLA-4	Martinez Chanza (33)	Ps (23%), Thyroiditis (13%), RA (11%)	106	35%	NA	NA	58% (NA)	36% (6%)	38% (12%)	6% and 8% permanent discontinuation due to AID flare and newly developed irAEs, respectively
Various	PD-1	Danlos (34)	Vitiligo (38%), Ps (27%), Thyroiditis (16%)	45	38%	NA	NA	44% (11%)	24% (NA)	22% (NA)	11% due to TirAEs
Various	PD-1	Cortellini (35)	Ps (40%), RA (27%), IBD (13%)	15^a^	50%	6.8	9.8	73% (13%)	47% (13%)	NA	13% due to TirAEs
			Thyroiditis (76%), Ps (10%), Vitiligo (3%)	70^b^	38%	14.4	15.7	64% (9%)	47% (9%)	NA	6% due to TirAEs
Various	PD-1 or CTLA-4	Tison (36)	Ps (28%), RA (18%), IBD (13%)	112	49%^c^	NA	NA	71% (NA)	47% (13%)	42% (16%)	21% permanent discontinuation due to TirAEs
Various	PD-1 or CTLA-4	Richter (37)	RA (31%), Polymyalgia rheumatica (31%), Sjogren’s syndrome (13%)	16	NA	NA	NA	38% (25%)	6% (0%)	31% (25%)	38% due to TirAEs
Various	PD-1 or CTLA-4	Kaur (38)	Hypothyroidism (61%), RA (9%), Ps (9%)	46	NA	NA	NA	NA	20% (NA)	NA	2% due to AID flare
Various	PD-1 or CTLA-4	Abu-Sbeih (39)	IBD (100%)	102	NA	NA	NA	NA	36% (17%)	NA	32% due to TirAEs
Various	PD-1 or CTLA-4	Braga Neto (40)	IBD (100%)	13	NA	NA	NA	NA	31% (NA)	NA	None
Various	PD-1 or CTLA-4	Efuni (41)	RA (100%)	22	NA	NA	NA	73%	55%	32% (9%)	9% and 5% permanent discontinuation due to AID flare and newly developed irAEs, respectively

AID, autoimmune disease; ANA, antinuclear antibody; CTLA-4, cytotoxic T lymphocyte-associated antigen 4; IBD, inflammatory bowel disease; irAEs, immune-related adverse events; mOS, median overall survival; mPFS, median progression free survival; NA, not available; NR, not reached; NSCLC, non–small cell lung cancer; ORR, objective response rate; PD-1, programmed cell death protein 1; Ps, psoriasis; RA, rheumatoid arthritis; TirAEs, total irAEs (i.e. AID flare and/or newly developed irAEs).

a Clinically active AID.

b Clinically inactive AID.

c In patients without prior ICI therapy.

### Efficacy and Safety of ICIs in Patients With Melanoma and AID

Rich experience has been accumulated in ICI therapy for malignant melanoma. There were three retrospective studies of anti-CTLA-4 therapy for melanoma patients with AID (26–28) (**Table 1**). Two studies reported that the objective response rate (ORR) was no more than 20% (26, 27), and another study, with just eight patients, reported an ORR as high as 50% (28). As to the safety, Johnson et al. (27) reported that the incidence of TirAEs in AID patients was 50%, including an incidence rate of AID flare of 27%. Kähler et al. (26) reported that the incidence of TirAEs in AID patients was 44%. The incidence of both AID flare and newly developed irAEs were 29%, and 17% of patients discontinued ICIs because of AID flare. Both studies (26, 27) supported that the incidence of TirAEs in AID patients did not exceed that observed in the general population included in previous large clinical trials (42, 43). Furthermore, AID flares and newly developed irAEs were usually manageable according to established algorithms (44–46), mostly with corticosteroids or other immunosuppressants, and did not preclude clinical benefit.

There were also two retrospective studies (15, 29) on anti-PD-1 therapy for melanoma patients with AID (**Table 1**). Both Menzies et al. (15) and Gutzmer et al. (29) reported that the ORR of anti-PD-1 antibodies in melanoma patients with preexisting AID exceeded 30%. Intriguingly, large clinical trials demonstrated that ORR of anti-PD-1 therapy in melanoma was 33% to 45% in the first-line treatment (42, 43, 47) and 21% to 32% after ipilimumab (48, 49). Considering the high prevalence of adverse prognostic features (such as 31% of patients had brain metastases, 48% of patients had elevated serum LDH, and 56% of patients’ ECOG grade were ≥1) and 54% of patients had previous anti-CTLA-4 therapy in the included patients, Menzies et al. (15) speculated that patients with a tendency toward autoimmunity might be more likely to benefit from anti-PD-1 therapy. Taken together, the above two studies (15, 29) suggested that the efficacy of anti-PD-1 therapy in patients with melanoma and AID was not inferior to that in the general population. As to the safety, Gutzmer et al. (29) reported that the incidence of any grade and grade 3/4 TirAEs were as high as 58% and 16%, respectively. The discontinuation rates of anti-PD-1 therapy in the study of Menzies et al. (15) were comparable to those in general melanoma patients (42, 43, 47), and the immune damage of AID flare was generally mild and manageable.

### Efficacy and Safety of ICIs in Patients With Other Cancers and AID

There are relatively few studies on ICI therapy in patients with non–small cell lung cancer (NSCLC) and preexisting AID. Yoneshima et al. (30) found that antinuclear antibody (ANA) positivity had no significant effect on ORR and the incidence of irAEs in NSCLC patients treated with anti-PD-1, but both the median progression-free survival (mPFS) and median overall survival (mOS) were significantly shorter in ANA-positive patients than in ANA-negative patients (2.9 *versus* 3.8 months, 11.6 *versus* 15.8 months, *p* = 0.03 for each instance). Moreover, the authors also identified three patients with increased ANA titer during the anti-PD-1 treatment, all of whom subsequently developed irAEs. The study of Leonardi et al. (31) demonstrated that the ORR was 22% in NSCLC patients with AID, the incidence of TirAEs was 55%, and the safety was comparable to that in the general population.

There were 141 patients with urological cancers and AID in the studies reported by Martinez Chanza et al. (33) and Loriot et al. (32). The most common preexisting AIDs were psoriasis (Ps, n = 39), thyroiditis (n = 30), and RA (n = 16). In the studies reported by Martinez Chanza et al. (33), the rates of AID flare and newly developed irAEs were as high as 36% and 38%, respectively. However, TirAEs in the above two studies (58% and 46%, respectively) were generally mild and reversible, especially in patients with asymptomatic or mildly symptomatic AID, and the efficacy was similar in AID and non-AID patients. As to AIDs of clinical concern, such as Guillain-Barre syndrome (GBS), MS, and IBD, flares did not appear more frequent but might be more aggressive as most of them resulted in ICI discontinuation.

In the studies on the use of ICIs in cancer patients with unlimited tumor types and preexisting AID, the majority of malignant tumor types were still melanoma and/or NSCLC (**Table 1**) (34–40) Danlos et al. (34) analyzed data from a large prospective study of anti-PD-1 treatment and found that the 45 patients with AID had no significant difference in ORR or mOS compared with those without AID, but the median irAE-free survival time was significantly shorter (5.4 *versus* 13.0 months, *p* = 0.0002) and the incidence of TirAEs was higher in patients with AID (44% *versus* 29%). Tison et al. (36) reported a large multicenter retrospective study including 112 AID patients treated for various cancers with ICIs. Forty-nine percent of 105 patients without prior ICI therapy were considered to be responders. After a median follow-up period of 8 months, the mPFS of melanoma and NSCLC patients was 12.9 months and 11.8 months, respectively, and the mOS was not reached and 22.4 months. However, the incidences of TirAEs and permanent treatment discontinuation (71% and 21%, respectively) were higher than that of the general population, which needs to be noticed. In the study of Cortellini et al. (35), the ORR of anti-PD-1 treatment in 85 cancer patients with AID was 40%. Unfortunately, the incidence of TirAEs in both inactive and active AID patients were significantly higher than that of the general population (64% *versus* 40%, *p* = 0.0005, and 73% *versus* 40%, *p* = 0.0162, respectively). The incidence of TirAEs is alarming and implies that clinicians should be vigilant when using ICIs in AID patients, especially in those with active AID. Nevertheless, the AID comorbidity in this study was not significantly related to the incidence of grade 3/4 irAEs, treatment discontinuation rate, ORR, PFS, or OS.

Summarily, the above studies tend to support the conclusion that the efficacy of ICIs in patients with AID is comparable to that in the general population, while the incidence of TirAEs is higher, most TirAEs are mild and manageable.

### The Effect of AID Status and Immunosuppressive Therapy on Efficacy and Safety of ICIs

It is controversial whether the use of glucocorticoids at a daily dose of 10 mg, or more of prednisone or other immunosuppressants, at the beginning of ICI therapy or within 1 month would affect the efficacy of ICI (50, 51). In the study of Kähler et al. (26), 11 of the 41 patients were undergoing at least one systemic immunosuppression at the time of ipilimumab initiation, including eight patients receiving a low-dose prednisone. The result suggested that patients receiving immunosuppressive therapy had a similar ORR of 9% compared to patients without immunosuppressive therapy. The studies of Gutzmer et al. (29) and Leonardi et al. (31) also supported that the use of immunosuppressants at the beginning of treatment does not affect the efficacy of ICI. However, in the study reported by Menzies et al. (15), 20 of the 52 patients with AID were on systemic immunosuppression at the start of anti-PD-1 therapy, including 14 patients receiving corticosteroids. The result showed that the ORR in the patients undergoing immunosuppressive therapy was significantly lower than in those not undergoing immunosuppressive therapy (15% *versus* 44%, *p* = 0.033). The meta-analysis of Xie et al. (52) also demonstrated that there was a trend toward lower ORR in patients with immunosuppressants at ICI start (*p* > 0.05). However, due to the heterogeneity of included patients, the data on the pooled ORR may not be so meaningful. As for survival data, Tison et al. (36) reported that the 51 patients receiving immunosuppressive therapy had a shorter mPFS than those without immunosuppressive agents (3.8 *versus* 12 months, *p* = 0.006), but the mOS difference was not significant. Furthermore, Cortellini et al. (35) reported that the patients with active AID tended to have a higher ORR but shorter mPFS and mOS when compared with patients with inactive AID.

On the aspect of safety, Menzies et al. (15) reported that flares occurred more often in patients with active AID than in those with inactive AID (60% *versus* 30%, *p* = 0.039), and there was a trend for more flares in patients on immunosuppression treatment than in those not on immunosuppression (50% *versus* 31%, *p* > 0.05). The study of Martinez Chanza et al. (33) also reached a conclusion consistent with that of Menzies et al. Similarly, the study of Leonardi et al. (31) suggested that AID was more likely to flare in patients with active symptoms, and Kähler et al. (26) also reported that flares occurred more often in patients on immunosuppression at the start of treatment. However, Abu-Sbeih et al. (39) reported that there was no significant correlation between active IBD within 3 months before ICIs initiation and all grades of gastrointestinal (GI) adverse events, but patients with active IBD had more severe GI adverse events. The meta-analysis of Xie et al. (52) reported that the pooled incidence of AID flare and newly developed irAEs were 35% and 33%, respectively, and there was no statistical difference between patients with and without immunosuppressive therapy regarding AID flare. Besides, almost all AID flares occurred at the beginning of ICI therapy—2 weeks to 1 year after initiation, but mostly around 1–2 months (15, 27, 29, 31, 33, 41). Most AID flares had similar manifestations and affected the same anatomic sites as prior AID symptoms (27, 31, 36).

Summarily, available studies mainly supported that immunosuppressive therapy controlling AID might have little influence on the efficacy of ICIs, but the incidence of TirAEs or AID flare would be higher in patients with active AID or undergoing immunosuppressive therapy. Furthermore, it is important to note that there are few patients with severe active AID included in the retrospective studies. It may be necessary to properly treat and control severe active AID before ICI initiation for safety’s sake.

### The Effect of AID and ICI Category on the Safety of ICIs

Almost all common AIDs have been included in the retrospective studies. However, some AIDs (such as neurological AIDs) will result in devastating consequences if they flare, so clinicians would rarely use ICIs in patients with those AIDs. Therefore, patients with those AIDs may be under-represented in the retrospective studies. Many studies yielded similar conclusions (15, 26–29, 31, 33–36, 38). That is, preexisting rheumatologic AIDs (such as RA, polymyalgia rheumatica) and Ps were most likely to effect AID flare after ICI therapy (the incidences of AID flare were 50% to 68% and 20% to 67% in patients with rheumatologic AIDs and Ps, respectively). This is in line with the review of Ramos-Casals et al. (53) and the meta-analysis of Xie et al. (52). Most of the studies suggested that autoimmune thyroiditis, GI AIDs (such as IBD/colitis), neurological AIDs, and respiratory AIDs (such as asthma) were less likely to effect AID flare (15, 26, 29, 31, 33, 34). Part of the possible reason for this is that the pathogenesis of various AIDs is heterogeneous. The PD-1/L1 pathway plays an important role in rheumatism, while many other AIDs do not involve or rely heavily on the PD-1-signaling pathway (15). For example, GBS is a typical B-cell-mediated disease, while chronic inflammation of RA is characterized by PD-1-positive T-cell infiltration (54, 55). However, due to a higher mortality rate in neurologic irAEs, NCCN guidelines had recommended more aggressive measures to neurological irAEs (46). Therefore, ICIs should be used more cautiously in patients with preexisting neurological AID.

As to ICI category, Tison et al. (36) reported that newly developed irAEs appeared to be more frequent (11/14 [79%] *versus* 34/95 [36%]) and severe in the ipilimumab group than in the anti-PD-1 therapy group. This is consistent with the conclusion that the incidence and severity of irAEs induced by anti-CTLA-4 treatment were higher or severer than that of anti-PD-1 treatment in the general population (56). This is also in line with severe autoimmunity and lymphoproliferative disorder observed in CTLA-4 gene deletion mice (18, 19) compared to moderate autoimmunity, including aplastic anemia, glomerulonephritis, and arthritis, seen in PD-1-deficient mice (57, 58). Colitis and hypophysitis were two common ipilimumab-induced irAEs in AID patients (26–28, 37). It is still not entirely clear why ICIs at different targets could produce organ-specific irAEs, and CTLA-4 expression on normal pituitary cells might explain hypophysitis induced by anti-CTLA-4 therapy (59, 60).

Colitis is a particularly frequent and potentially fatal ipilimumab-induced irAE, so the use of ipilimumab in IBD patients was of particular clinical interest. The study reported by Abu-Sbeih et al. (39) enrolled 102 IBD patients treated with ICIs, 17 of those patients receiving ipilimumab. The results showed that anti–CTLA-4 therapy and IBD involving the colon before ICIs initiation were possible risk factors for GI toxicities. However, no GI adverse event–related death was reported, and the response to ICIs in patients with underlying IBD was comparable to that in non-IBD patients, indicating the potential clinical benefit. The study reported by Johnson et al. (27) included six asymptomatic or minimally symptomatic IBD patients receiving ipilimumab. Three patients had prior colectomies, and the other three patients were receiving aminosalicylate derivatives or topical hydrocortisone at the time of ipilimumab initiation. After ipilimumab treatment, one patient with ulcerative colitis developed an AID flare and another patient with Crohn disease had newly developed ipilimumab-induced colitis; these were resolved by infliximab and corticosteroids, respectively. There were three IBD patients receiving ipilimumab in the study of Kähler et al. (26), and two of them were receiving mesalazine at the time of ipilimumab initiation. Only one patient with ulcerative colitis developed an AID flare, and this was resolved after treatment discontinuation and prednisone pulse therapy. Therefore, we suggest that ipilimumab might be considered for cancer patients with IBD, if necessary, provided that IBD status is stable and closely monitored.

A systematic review conducted by Abdel-Wahab et al. (61) in 2018 included 123 patients with preexisting AID receiving ICIs. The results demonstrated that no differences in irAEs were observed in patients with active *versus* inactive AID, and patients receiving immunosuppressive therapy at the time of ICIs initiation appeared to have fewer irAEs. In this systematic review, the incidence of AID flare induced by anti-PD-1 was higher than that by ipilimumab, and more newly developed irAEs were reported with ipilimumab. Some conclusions reached by this systematic review were contradictory to other reviews (6, 56), including ours. This may be partly due to the small number of cases and the variety of AID included in the study of Abdel-Wahab et al., so prospective studies are needed to determine the safety and efficacy of ICIs in patients with AID (6).

## Strategies to Prevent and Manage irAEs in AID Patients

Immunotherapy-induced inflammation and tumor lysis generate numerous antigens that can be presented by antigen-presenting cells and trigger a secondary immune response to autoantigen. This process is defined as epitope spreading, which plays a crucial role in the initial stage of irAE development (62). Multiple studies demonstrated that the incidence of irAEs is significantly associated with the efficacy of ICIs (63), which can be partly explained by epitope spreading. Epitope spreading can also explain why different tumors treated with ICIs are associated with specific irAEs. For instance, in terms of antigenic epitopes, skin is the organ that shares T cell antigens with NSCLC only second to the lung and had the second highest incidence of irAEs (64). Berner et al. (64) identified nine shared T cell antigens in the lung tumor and skin in 25 patients with ICIs-induced skin toxicity. Besides, melanoma patients were more likely to develop mucosal and dermatological toxicities (65). Therefore, the use of ICIs should be prudent in melanoma or NSCLC patients with dermatological AIDs. Moreover, as mentioned above, some AIDs were inclined to worsen or result in devastating consequences during ICI therapy. Hence, in these specific situations, such as RA or GBS with active disease status, ICI applications should be avoided as much as possible. Furthermore, there have been many studies on biomarkers predicting irAEs (66), and increased autoantibody titers can also detect irAEs early (30, 67, 68). Therefore, we do not think it is safe enough to use ICIs in patients with biomarkers that indicate a predisposition to specific irAEs in the same organs involved by their AID. For example, in a study of 26 melanoma patients treated with anti-CTLA-4, Firmicutes were associated with ICIs-induced colitis (69). Therefore, we should be more careful if we use ICIs in IBD patients with increased Firmicutes in the gut microbiome. However, those biomarkers predicting irAEs need to be validated prospectively.

To lower the risk of compromising ICIs efficacy, corticosteroids should be avoided as much as possible at the time of ICIs initiation. However, it is necessary to properly treat and control the preexisting AID before ICIs initiation. Therefore, Haanen et al. (70) proposed a two-step strategy for managing irAEs in AID patients. First, non-selective immunosuppressants (such as corticosteroids and mycophenolate mofetil) could be replaced by specific selective immunosuppressants (such as tocilizumab [anti-IL-6 receptor antibody], infliximab [anti-TNF-α antibody], and vedolizumab [anti-α4β7 integrin antibody]) to control AIDs for a short period. Subsequently, combining ICIs with selective immunosuppressants could prevent the flare of preexisting AID. In a study of 87 patients who developed irAEs after treatment with nivolumab, clinical improvement was observed in 79% of 34 patients who received anti-IL-6 receptor antibody tocilizumab (71). On the aspect of efficacy, inhibition of IL-6 has been reported to have synergistic anti-tumor activity when combined with ICIs in mouse models (65). Small series and several cases also have reported the safe and effective administration of ICIs in active AIDs under selective immunosuppressants (70). Theoretically, the two-step strategy recommended by Haanen et al. (70) seems to be valid when administrating ICIs in AID patients. Nevertheless, it is also important to note that the strategy is based on small-scale retrospective studies. Further studies are needed to confirm the effectiveness and safety of this strategy.

Intriguingly, irAEs can be prevented to some extent by modifying ICIs so that they are only active within the TME, or restricting their delivery to the TME. For example, ICIs can be loaded onto nanoparticles which possess intrinsic properties, like magnetism, and then be actively directed to accumulate in TME with the help of external devices (72). However, biomaterial-based cancer immunotherapies are mainly explored in animal studies, and deserve further validation in clinical trials.

## Conclusion

There is no available prospective study on whether the efficacy and safety of ICIs in patients with cancer and preexisting AID differ from that in the general population. The results of the existing retrospective studies seemed to support that the efficacy of ICIs in patients with preexisting AID was comparable to that in the general population, and while the incidence of irAEs was higher, most irAEs were mild and manageable. However, there is a selection bias that most of the AID patients included in the existing retrospective studies were in stable disease status, patients’ performance status was fair, and certain AIDs inclined to result in devastating consequences during ICI therapy may be under-represented. Given the existing data, preexisting AID may not be an absolute contraindication to ICI therapy. It is recommended to weigh the benefits and risks of immunotherapy in a multidisciplinary approach including rheumatologists based on a patient’s AID category and severity, the patient’s cancer type and prognosis, alternative treatment options, and the patient’s intention. After that, ICIs could be administered with close monitoring of irAEs in patients with good performance status and mild AID status who could benefit from immunotherapy. Moreover, there are several strategies for preventing and meagering irAEs that can be taken when administrating ICIs in AID patients. Two phase I clinical trials of nivolumab in patients with AID and lung cancer (NCT03656627) or across tumor types (NCT03816345) are ongoing. The results of the above two trials and more real-world retrospective studies would reach more instructive conclusions about the existing controversies.

## Author Contributions

Conception and design: HT and JZ. Literature review and analysis: HT and JZ. Drafting of the manuscript: HT and JZ. Supervision: JZ and CB. All authors contributed to the article and approved the submitted version.

## Funding

This work is supported by a grant from CAMS Innovation Fund for Medical Sciences (no. 2016-I2M-1-001).

## Conflict of Interest

The authors declare that the review was conducted in the absence of any commercial or financial relationships that could be construed as a potential conflict of interest.
